# Prevalence of Diagnosed Arthritis — United States, 2019–2021

**DOI:** 10.15585/mmwr.mm7241a1

**Published:** 2023-10-13

**Authors:** Elizabeth A. Fallon, Michael A. Boring, Anika L. Foster, Ellen W. Stowe, Tyler D. Lites, Erica L. Odom, Puja Seth

**Affiliations:** ^1^Division of Population Health, National Center for Chronic Disease Prevention and Health Promotion, CDC; ^2^American Society of Radiologic Technologists, Smyrna, Georgia; ^3^Oak Ridge Institute for Science and Education, Oak Ridge, Tennessee.

**Summary**

**What is already known about this topic?**

Arthritis is a leading cause of activity limitations, disability, and chronic pain, and is associated with dispensed opioid prescriptions, substantially contributing to health care costs.

**What is added by this report?**

During 2019–2021, 21.2% of U.S. adults (53.2 million) reported diagnosed arthritis. Approximately one half (52.2%–62.4%) of adults aged ≥65 years with self-reported diagnosed dementia, chronic obstructive pulmonary disease, stroke, heart disease, diabetes, or cancer also had a reported diagnosis of arthritis.

**What are the implications for public health practice?**

These prevalence estimates can be used to guide public health policies and activities to increase equitable access to physical activity opportunities within the built environment and other community-based, arthritis-appropriate, evidence-based interventions.

## Abstract

Arthritis includes approximately 100 conditions that affect the joints and surrounding tissues. It is a leading cause of activity limitations, disability, and chronic pain, and is associated with dispensed opioid prescriptions, substantially contributing to health care costs. Combined 2019–2021 National Health Interview Survey data were analyzed to update national prevalence estimates of self-reported diagnosed arthritis. An estimated 21.2% (18.7% age-standardized) of U.S. adults aged ≥18 years (53.2 million) had diagnosed arthritis during this time frame. Age-standardized arthritis prevalences were higher among women (20.9%) than men (16.3%), among veterans (24.2%) than nonveterans (18.5%), and among non-Hispanic White (20.1%) than among Hispanic or Latino (14.7%) or non-Hispanic Asian adults (10.3%). Adults aged ≥45 years represent 88.3% of all U.S. adults with arthritis. Unadjusted arthritis prevalence was high among adults with chronic obstructive pulmonary disease (COPD) (57.6%), dementia (55.9%), a disability (54.8%), stroke (52.6%), heart disease (51.5%), diabetes (43.1%), or cancer (43.1%). Approximately one half of adults aged ≥65 years with COPD, dementia, stroke, heart disease, diabetes, or cancer also had a diagnosis of arthritis. These prevalence estimates can be used to guide public health policies and activities to increase equitable access to physical activity opportunities within the built environment and other arthritis-appropriate, evidence-based interventions.

## Introduction

Arthritis includes approximately 100 conditions that affect the joints and surrounding tissues. If not managed properly, arthritis can result in severe pain, activity limitations, and disability (*1*–*3*). Adults with arthritis have disproportionate rates of anxiety and depression and received 55.3% of all-cause prescription opioids dispensed in the United States in 2015 (*4*,*5*). Thus, arthritis is a significant driver of lost wages, disability, and medical costs (*3*,*6*). Updated arthritis prevalence estimates can help identify disproportionately affected groups, monitor arthritis prevalence over time, and guide resource allocation.

## Methods

The National Health Interview Survey (NHIS) is an annual, nationally representative household survey of the noninstitutionalized U.S. civilian population.* One adult in a household is randomly selected to complete the in-home interview. When necessary, telephone follow-up is permitted to complete the interview.^†^ If the selected person is physically or mentally unable to answer the survey, a knowledgeable proxy can answer on behalf of the selected person. During April–June 2020, the COVID-19 pandemic necessitated a change to telephone-only data collection. During July 2020–April 2021, interviews were attempted by telephone first, with in-home follow-up to complete data collection. In May 2021, data collection returned to prepandemic procedures.^§^

NHIS sample sizes and response rates for 2019, 2020, and 2021 were 31,997 (59.1%), 21,153 (48.9%), and 29,482 (50.9%), respectively.^¶^ A person was identified as having arthritis if he or she responded “yes” to the question, “Have you ever been told by a doctor or other health care professional that you have arthritis, rheumatoid arthritis, gout, lupus, or fibromyalgia?” Survey respondents answering “yes” or “no” to this item were included in the analytic sample (82,503; 99.8% of survey respondents). Data were weighted to account for complex survey design, selection probability, and nonresponse. Unadjusted and age-standardized** arthritis prevalence estimates for adults aged ≥18 years were calculated overall and by selected self-reported demographic and health characteristics. Subgroup prevalences were compared with a reference group using t-tests; all differences are significant at α = 0.05. Analyses were conducted in SAS (version 9.4; SAS Institute) and SUDAAN (version 11.0; RTI International). This activity was reviewed by CDC, deemed not research, and was conducted consistent with applicable federal law and CDC policy.^††^

## Results

Approximately 53.2 million (95% CI = 52.1–54.4) or 21.2% (18.7% age-standardized) of U.S. adults aged ≥18 years had diagnosed arthritis (Table 1). Age-standardized prevalence was higher among women (20.9%) than men (16.3%) and among veterans (24.2%) than nonveterans (18.5%). Age-standardized prevalence was higher among non-Hispanic White (20.1%) than among Hispanic or Latino (14.7%) or non-Hispanic Asian adults (10.3%). There was no difference between non-Hispanic White and non-Hispanic Black or African American adults (19.7%), or non-Hispanic American Indian or Alaska Native adults (21.0%). The unadjusted prevalence of arthritis among U.S. adults with a disability was 54.8% (12.3 million); after adjusting for age, prevalence was higher among adults with a disability (40.5%) than among those without a disability (16.6%).

**TABLE 1:** Unadjusted and age-standardized* prevalence of diagnosed arthritis^†^ among adults aged ≥18 years, by selected characteristics — National Health Interview Survey, United States, 2019–2021

Characteristic	Unweighted no. with arthritis^§^	Weighted no. with arthritis (millions)^§^	Distribution among adults with arthritis (%)^¶^	Prevalence of arthritis^†^	
				Unadjusted % (95% CI)	Age-standardized % (95% CI)
**Overall (2019–2021)**	**21,204**	**53.2**	**100**	**21.2 (20.7–21.6)**	**18.7 (18.3–19.1)**
**Survey year**					
2019 (Ref)	8,214	53.6	NA	21.4 (20.8–22.0)	19.2 (18.7–19.7)
2020**	5,382	52.3	NA	20.8 (20.0–21.5)	18.3 (17.7–19.0)**^††^**
2021	7,608	53.8	NA	21.3 (20.7–21.9)	18.6 (18.1–19.1)
**Age group, yrs**					
18–44 (Ref)	1,905	6.2	11.7	5.4 (5.1–5.7)	NA
45–64	7,390	21.3	40.0	26.0 (25.3–26.8)^§§^	NA
≥65	11,880	25.7	48.3	47.3 (46.4–48.2)^§§^	NA
**Sex^¶¶^**					
Female	13,032	31.5	59.2	24.2 (23.6–24.9)^§§^	20.9 (20.5–21.4)^§§^
Male (Ref)	8,171	21.7	40.8	17.9 (17.3–18.4)	16.3 (15.8–16.7)
Race and ethnicity***					
American Indian or Alaska Native, NH	153	0.4	0.7	22.2 (17.3–28.1)	21.0 (16.8–26.0)
Asian, NH	496	1.6	2.9	10.5 (9.3–11.8)^§§^	10.3 (9.3–11.4)^§§^
Black or African American, NH	2,349	6.0	11.3	20.4 (19.3–21.6)^§§^	19.7 (18.8–20.7)
White, NH (Ref)	16,236	39.1	73.3	24.6 (24.1–25.2)	20.1 (19.7–20.6)
Hispanic or Latino, any race	1,586	5.2	9.8	12.4 (11.6–13.2)^§§^	14.7 (13.9–15.4)^§§^
Other, multiple races, NH	384	1.0	1.9	20.8 (18.3–23.6)^§§^	23.6 (21.1–26.2)^§§^
**Sexual orientation^†††^**					
Bisexual	201	0.6	1.1	12.6 (10.4–15.2)^§§^	24.7 (21.1–28.6)^§§^
Lesbian, gay, or homosexual	285	0.7	1.3	16.1 (14.0–18.4)^§§^	18.4 (16.4–20.5)
Straight or heterosexual (Ref)	19,755	49.4	96.2	21.5 (21.0–22.0)	18.6 (18.3–19.0)
Something else	90	0.2	0.5	20.0 (15.6–25.3)	25.6 (21.0–30.8)^§§^
Don't know	184	0.5	0.9	18.6 (15.3–22.3)	18.1 (15.3–21.3)
**Highest educational attainment^§§§^**					
Less than high school graduate (Ref)	2,380	7.4	14.0	26.3 (25.0–27.6)	20.3 (19.3–21.4)
High school graduate or equivalent	6,021	16.0	30.3	23.1 (22.3–23.9)^§§^	20.2 (19.5–20.8)
At least some college	6,585	16.2	30.6	22.0 (21.2–22.8)^§§^	20.3 (19.7–20.9)
College degree or greater	6,099	13.2	25.1	16.8 (16.3–17.4)^§§^	15.3 (14.8–15.7)^§§^
**Employment status^¶¶¶^**					
Employed or self-employed (Ref)	6,893	19.3	37.4	12.6 (12.2–13.1)	14.8 (14.4–15.2)
Retired	10,041	21.7	42.2	47.4 (46.4–48.3)^§§^	30.1 (23.5–37.5)^§§^
Unable to work or disabled	2,732	7.4	14.4	49.1 (47.4–51.0)^§§^	40.9 (39.0–42.9)^§§^
Unemployed	228	0.8	1.5	11.2 (9.6–13.1)	16.5 (14.1–19.3)
Other	730	2.4	4.6	10.2 (9.3–11.2)^§§^	16.1 (14.8–17.5)
Income to poverty ratio****					
Poor/Near poor (<125%; Ref)	3,811	8.5	17.3	25.6 (24.5–26.7)	25.1 (24.1–26.0)
Low income (125% to <200%)	3,384	7.7	15.7	23.5 (22.4–24.6)^§§^	20.6 (19.7–21.6)^§§^
Middle income (200% to <400%))	6,465	15.3	31.3	22.0 (21.3–22.7)^§§^	19.5 (18.9–20.1)^§§^
High income (≥400%)	7,544	17.4	35.7	18.2 (17.7–18.8)^§§^	15.7 (15.3–16.1)^§§^
**Veteran status** ^††††^					
Yes (Ref)	2,916	6.8	13.2	35.2 (33.9–36.6)	24.2 (22.9–25.7)
No	17,716	44.7	86.8	20.0 (19.5–20.4)^§§^	18.5 (18.1–18.9)^§§^
**BMI (kg/m^2^)^§§§§^**					
Underweight/Healthy weight (<25; Ref)	5,396	12.7	24.6	15.5 (15.0–16.1)	14.7 (14.2–15.2)
Overweight (25 to <30)	6,852	17.1	33.0	20.5 (19.9–21.1)^§§^	16.8 (16.3–17.3)^§§^
Obesity (≥30)	8,373	22.0	42.4	27.5 (26.7–28.3)^§§^	24.5 (23.9–25.2)^§§^
**Any disability^¶¶¶¶^**					
Yes (Ref)	5,112	12.3	23.0	54.8 (53.4–56.2)	40.5 (38.9–42.2)
No	16,092	41.0	77.0	17.9 (17.4–18.3)^§§^	16.6 (16.3–17.0)^§§^
Heart disease*****					
Yes (Ref)	3,516	8.2	15.5	51.5 (49.9–53.1)	36.3 (32.9–39.8)
No	17,603	44.8	84.5	19.0 (18.6–19.5)^§§^	17.8 (17.4–18.2)^§§^
**Diabete**s*****					
Yes (Ref)	4,072	10.2	19.1	43.1 (41.8–44.5)	28.8 (27.1–30.5)
No	17,117	43.0	80.9	18.9 (18.4–19.3)^§§^	17.8 (17.4–18.1)^§§^
Cancer*****					
Yes (Ref)	3,641	8.3	16.3	43.1 (41.5–44.6)	27.7 (25.8–29.8)
No	16,511	42.6	83.7	18.8 (18.3–19.2)^§§^	18.0 (17.6–18.3)^§§^
Dementia*****					
Yes (Ref)	542	1.4	2.6	55.9 (52.0–59.7)	47.8 (36.7–59.1)
No	20,645	51.8	97.4	20.8 (20.3–21.3)^§§^	18.6 (18.2–19.0)^§§^
Stroke*****					
Yes (Ref)	1,563	3.7	7.0	52.6 (50.3–55.0)	39.0 (34.6–43.7)
No	19,619	49.5	93.0	20.2 (19.8–20.7)^§§^	18.3 (17.9–18.7)^§§^
COPD*****					
Yes (Ref)	2,818	6.8	12.9	57.6 (55.8–59.5)	43.5 (40.8–46.2)
No	18,356	46.3	87.1	19.3 (18.9–19.8)^§§^	17.6 (17.2–17.9)^§§^

**Abbreviations:** BMI = body mass index; COPD = chronic obstructive pulmonary disease; NA = not applicable; NH = non-Hispanic; Ref = referent group.

* Unadjusted and age-standardized estimates are calculated using sampling weights to produce nationally representative prevalences. Age-standardized estimates were calculated using 82,290 (99.6%) respondents, with complete data for both arthritis and age questions. Estimates are age-standardized to the 2000 U.S. projected adult population, using three age groups: 18–44, 45–64, and ≥65 years. https://www.cdc.gov/nchs/data/statnt/statnt20.pdf

^†^ Responded “yes” to the question, “Have you ever been told by a doctor or other health professional that you had some form of arthritis, rheumatoid arthritis, gout, lupus, or fibromyalgia?”

^§^ Might not sum to column total for some categories because of item-specific missing data.

^¶^ Might not sum to 100% because of rounding.

** During the COVID-19 pandemic (April 2020–May 2021), data collection procedures changed from in-home to telephone-based and back to in-home. This analysis combined the 2020 partial adult sample data file with 2019 and 2021 data to obtain arthritis prevalence for 2019–2021. Therefore, the 2020 estimate displayed is only used to explore the potential effects of COVID-19 and should not be used as a single year estimate of diagnosed arthritis for 2020. https://ftp.cdc.gov/pub/Health_Statistics/NCHS/Dataset_Documentation/NHIS/2020/srvydesc-508.pdf

**^††^** T-tests were used to determine statistically significant differences in arthritis prevalence by survey year; differences with p≤0.05 were considered statistically significant. Despite statistically significant differences by year for age-adjusted arthritis prevalence, crude prevalence and weighted population estimates were not substantively different across years.

^§§^ T-tests were used to determine statistically significant differences in arthritis prevalence for subgroups defined by selected characteristics; differences with p≤0.05 were considered statistically significant.

^¶¶^ Identified as “male” or “female” in response to the question, “are you male or female?”

*** Racial and ethnic categories from the sample adult file codebook were used. Persons identifying as “other single and multiple race,” or “NH American Indian or Alaska Native and any other group” were combined into “Other, multiple races, NH.”

^†††^ The sample adult file codebook sexual orientation categories were used. Survey responses coded as “refused,” “don’t know,” “not ascertained,” or missing responses were excluded.

^§§§^ Responses to the question, “What is the highest level of school you completed…?” were combined into the following groups: 1) less than high school graduate: never attended/attended only kindergarten, grades 1–11, or attended 12th grade but did not get a diploma, 2) high school graduate or equivalent: graduated high school, or earned a general education development certificate, 3) at least some college: some college or associate degree, and 4) college degree or greater: bachelor’s, master’s, professional school, or doctoral degree.

^¶¶¶^ Persons responding “yes” to the question, “Do you usually work 35 hours or more per week in total at all jobs or businesses?” were considered “employed/self-employed.” Persons responding “no” were asked “What is the MAIN reason you were not working for pay…?” with responses categorized as 1) unemployed: “unemployed, laid off, seasonal/contract work, looking for work,” 2) retired: “retired,” 3) unable to work/disabled: “unable to work for health reasons/disabled,” and 4) other: all remaining valid options.

**** Income-to-poverty ratio values for the income-to-poverty ratio variable were calculated using National Health Interview Survey imputed income files. https://ftp.cdc.gov/pub/Health_Statistics/NCHS/Dataset_Documentation/NHIS/2021/NHIS2021-imputation-techdoc-508.pdf; https://ftp.cdc.gov/pub/Health_Statistics/NCHS/Dataset_Documentation/NHIS/2020/NHIS2020-imputation-techdoc-508.pdf; https://ftp.cdc.gov/pub/Health_Statistics/NCHS/Dataset_Documentation/NHIS/2019/NHIS2019-imputation-techdoc-508.pdf

^††††^ Veterans were defined as respondents who answered “yes” to “Did you ever serve on active duty in the U.S. Armed Forces, military Reserves, or National Guard?”

^§§§§^ Self-reported height and weight were used to calculate BMI [weight (kg)/(height [m])^2^].

^¶¶¶¶^ A respondent who reported a lot of difficulty or being unable to do an activity in any of the six Washington Group Short Set on Functioning items (vision, hearing, mobility, self-care, cognitive, or communication) was considered to have a disability.

***** Respondents were considered as having the following chronic diseases if they answered “yes” to “Have you ever been told by a doctor or other health professional that you have/had…” 1) heart disease: coronary heart disease, angina (angina pectoris), or heart attack (myocardial infarction); 2) cancer: cancer or a malignancy of any kind (excluding skin cancer); 3) dementia: dementia, including Alzheimer disease; 4) COPD, emphysema, or chronic bronchitis, 5) stroke, or 6) diabetes.

Adults aged ≥45 years represent 88.3% of all U.S. adults with arthritis. Nearly one half of adults with arthritis (48.3%; 25.7 million) were aged ≥65 years, and 40.0% (21.3 million) were aged 45–64 years. Age-standardized prevalence of arthritis was higher among adults with dementia (47.8%),^§§^ chronic obstructive pulmonary disease (COPD) (43.5%), stroke (39.0%), heart disease (36.3%), diabetes (28.8%), or cancer (27.7%), than among adults without these chronic conditions (Table 1). The U.S. arthritis prevalence was substantial among adults with COPD (57.6%; 6.8 million), dementia (55.9%; 1.4 million), stroke (52.6%; 3.7 million), heart disease (51.5%; 8.2 million), diabetes (43.1%; 10.2 million), or cancer (43.1%; 8.3 million). Among adults aged ≥65 years, approximately one half of those with COPD (62.4%), stroke (57.9%), heart disease (57.4%), obesity (body mass index ≥30 kg/m^2^; 56.8%), dementia (56.1%), diabetes (54.0%), or cancer (52.2%) also had diagnosed arthritis (Table 2) (Figure).

**TABLE 2:** Prevalence* of diagnosed arthritis^†^ among adults aged ≥18 years with selected chronic conditions, by age group — National Health Interview Survey, United States, 2019–2021

Health condition	Age group, yrs		
	18–44 % (95% CI)	45–64 % (95% CI)	≥65 % (95% CI)
Obesity (BMI ≥30)^§^	8.6 (7.9–9.3)	34.4 (33.2–35.7)	56.8 (55.2–58.3)
Heart disease^¶^	23.7 (18.0–30.5)	46.6 (43.6–49.6)	57.4 (55.5–59.2)
Diabetes^¶^	15.0 (12.2–18.3)	38.8 (36.7–40.9)	54.0 (52.2–55.8)
Cancer^¶^	15.2 (12.1–18.8)	36.1 (33.6–38.8)	52.2 (50.4–54.0)
Dementia^¶^	—**	59.4 (48.3–69.5)	56.1 (51.9–60.2)
Stroke^¶^	26.8 (19.5–35.6)	50.0 (45.6–54.4)	57.9 (55.1–60.7)
COPD^¶^	26.8 (22.2–31.9)	62.4 (59.4–65.4)	62.4 (60.0–64.8)
No chronic condition^††^	3.6 (3.3–3.9)	17.1 (16.3–7.9)	36.7 (35.4–38.1)

**Abbreviations:** BMI = body mass index; COPD = chronic obstructive pulmonary disease.

* Calculated using sampling weights to produce nationally representative prevalence estimates.

^†^ Responded “yes” to the question, “Have you ever been told by a doctor or other health professional that you had some form of arthritis, rheumatoid arthritis, gout, lupus, or fibromyalgia?”

**^§^** Self-reported height and weight were used to calculate BMI [weight (kg)/(height [m])^2^].

^¶^ Respondents were considered as having the following chronic diseases if they answered “yes” to “Have you ever been told by a doctor or other health professional that you have/had…” 1) heart disease: coronary heart disease, angina (angina pectoris), or heart attack (myocardial infarction); 2) cancer: cancer or a malignancy of any kind (excluding skin cancer); 3) dementia: dementia, including Alzheimer disease; 4) COPD, emphysema, or chronic bronchitis, 5) stroke, or 6) diabetes.

** The estimate is suppressed on the basis of the data presentation standards for proportions. https://www.cdc.gov/nchs/data/series/sr_02/sr02_175.pdf

^††^ Respondents answered “no” to “Have you ever been told by a doctor or other health professional that you have/had…” for all of the following: 1) heart disease: coronary heart disease, angina (angina pectoris), or heart attack (myocardial infarction); 2) cancer: cancer or a malignancy of any kind (excluding skin cancer); 3) dementia: dementia, including Alzheimer disease; 4) COPD, emphysema, or chronic bronchitis, 5) stroke, or 6) diabetes.

**FIGURE:**
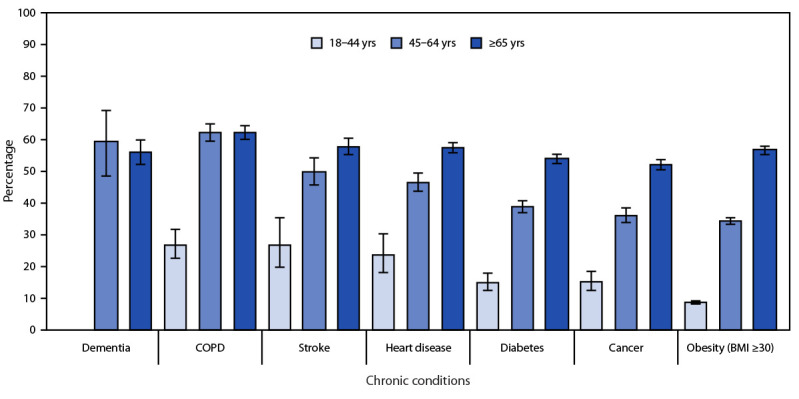
Prevalence*^,†^ of diagnosed arthritis^§^ among adults aged ≥18 years with selected chronic conditions,^¶^^,^** by age group — National Health Interview Survey, United States, 2019–2021 **Abbreviations**: BMI = body mass index; COPD = chronic obstructive pulmonary disease. * Calculated using sampling weights to produce nationally representative prevalence estimates. 95% CIs indicated by error bars. ^†^ The estimate for dementia (18–44 years) is suppressed based on the data presentation standards for proportions. https://www.cdc.gov/nchs/data/series/sr_02/sr02_175.pdf **^§^** Responded “yes” to the question, “Have you ever been told by a doctor or other health professional that you had some form of arthritis, rheumatoid arthritis, gout, lupus, or fibromyalgia?” ^¶^ Respondents were considered as having the following chronic diseases if they answered “yes” to “Have you ever been told by a doctor or other health professional that you have or had…” 1) heart disease: coronary heart disease, angina (angina pectoris), or heart attack (myocardial infarction); 2) cancer: cancer or a malignancy of any kind (excluding skin cancer); 3) dementia: dementia, including Alzheimer disease; 4) COPD, emphysema, or chronic bronchitis, 5) stroke, or 6) diabetes. ** Self-reported height and weight were used to calculate BMI [weight (kg)/(height [m])^2^].

## Discussion

During 2019–2021, 53.2 million (21.2%) U.S. adults aged ≥18 years had diagnosed arthritis. Approximately one half of adults aged ≥65 years with a chronic disease also reported diagnosed arthritis. Consistent with previous NHIS (*1*) and Behavioral Risk Factor Surveillance System data (*7*), arthritis prevalence was higher among women than men, among adults aged 45–64 and ≥65 years than among those aged 18–44 years, among veterans than among nonveterans, among persons with a disability than among those without a disability, and among persons reporting a comorbid chronic condition than among those without such a condition.

The 2019–2021 NHIS prevalence estimate is lower than the 2016–2018 NHIS prevalence estimate (58.5 million; 23.7%) (*1*). The NHIS survey was redesigned in 2019 (*8*), resulting in reordering and eliminating some arthritis-relevant questions, which might have led to differences in respondents’ ability to recall an arthritis diagnosis. Therefore, estimates produced by NHIS before 2019 should not be statistically compared with NHIS estimates after 2019, but instead interpreted as independent estimates obtained from different survey methodologies. This study establishes a new baseline for monitoring NHIS arthritis prevalence estimates, beginning with the combined 2019–2021 data. These estimates can be used to guide public health activities, policies, and resource allocation for improving arthritis-attributable health outcomes and associated health care costs.

### Limitations

The findings in this report are subject to at least five limitations. First, because of the cross-sectional nature of NHIS, causality among selected characteristics and arthritis diagnosis cannot be inferred. Second, arthritis diagnosis was self-reported and was not validated by medical record review. Third, social desirability, recall, and proxy response biases might lead to over- or underestimation of arthritis prevalence. Fourth, the single survey item assessing diagnosed arthritis does not capture undiagnosed arthritis or allow prevalence estimates to be calculated for arthritis subtypes. Finally, although annual prevalence estimates of arthritis were similar during 2019–2021, the COVID-19 pandemic necessitated changes in data collection methods and might have altered U.S. adult health care use and potentially affected estimates of diagnosed arthritis.

### Implications for Public Health Practice

Physical activity is important for managing arthritis-attributable pain and improving physical function (*9*). Many adults, including those with arthritis, do not meet the 2018 Physical Guidelines for Americans recommendations for physical activity despite its known benefits^¶¶^; these recommendations include engaging in 150–300 minutes of moderate-intensity or 75–150 minutes of vigorous-intensity activity per week or an equivalent combination, as well as ≥2 days of strength training per week. The guidelines also recommend that adults aged ≥65 years do multicomponent physical activity that includes balance training and aerobic and muscle strengthening activities. When an older adult or adult with arthritis cannot meet these recommendations because of health conditions, they need to be as physically active as their health and abilities allow.***

Maintaining a healthy weight, engaging in sufficient physical activity, and avoiding joint injury are important for prevention. The CDC Arthritis Management and Wellbeing Program recognizes community-based, arthritis-appropriate, evidence-based interventions (AAEBIs)^†††^ to increase physical activity and chronic disease self-management among adults with arthritis. A recommendation from a health care provider can increase the likelihood that adults with arthritis attend education programs and engage in physical activity (*10*). CDC funds national and state organizations^§§§^ to increase the availability of AAEBIs in the community, as well as to increase awareness among health care providers and health systems about the need to screen adults with arthritis for physical activity and facilitate the use of appropriate tools for physical activity screening, counseling, and referral to AAEBIs, with emphasis on reaching populations and communities with high prevalences of arthritis. Increasing equitable access to physical activity opportunities within the built environment and across settings (e.g., worksites, community organizations, and home), implementing AAEBIs as independent interventions or in combination with other chronic disease management programs (e.g., Diabetes Prevention Program), and adopting health care systems policies and actions facilitating health care provider screening, counseling, and referrals or linkages, are public health priorities to address arthritis and arthritis-attributable health outcomes.

## Footnotes

*
https://www.cdc.gov/nchs/nhis/index.htm

†
https://www.cdc.gov/nchs/nhis/data-questionnaires-documentation.htm

§ https://ftp.cdc.gov/pub/Health_Statistics/NCHS/Dataset_Documentation/NHIS/2019/srvydesc-508.pdf; https://ftp.cdc.gov/pub/Health_Statistics/NCHS/Dataset_Documentation/NHIS/2020/srvydesc-508.pdf; https://ftp.cdc.gov/pub/Health_Statistics/NCHS/Dataset_Documentation/NHIS/2021/srvydesc-508.pdf

¶ Per NHIS instructions, the “sample adult partial” data file from 2020 was combined with the 2019 and 2021 data for this analysis. https://www.cdc.gov/nchs/nhis/2020nhis.htm

** Age-standardized to the 2000 projected U.S. adult population with three age groups (18–44, 45–64, and ≥65 years). https://www.cdc.gov/nchs/data/statnt/statnt20.pdf

†† 45 C.F.R. part 46.102(l)(2), 21 C.F.R. part 56; 42 U.S.C. Sect. 241(d); 5 U.S.C. Sect. 552a; 44 U.S.C. Sect. 3501 et seq.

§§ A total of 45.9% of respondents with dementia used a survey proxy, compared with 2.0% of survey respondents without dementia, indicating greater potential for proxy response bias among adults with dementia.

¶¶ Part C. https://health.gov/sites/default/files/2019-09/PAG_Advisory_Committee_Report.pdf

*** https://health.gov/sites/default/files/2019-09/Physical_Activity_Guidelines_2nd_edition.pdf^†††^ https://www.cdc.gov/arthritis/interventions/index.htm

§§§
https://www.cdc.gov/arthritis/partners/index.htm
